# Three-dimensional solvation structure of ethanol on carbonate minerals

**DOI:** 10.3762/bjnano.11.74

**Published:** 2020-06-10

**Authors:** Hagen Söngen, Ygor Morais Jaques, Peter Spijker, Christoph Marutschke, Stefanie Klassen, Ilka Hermes, Ralf Bechstein, Lidija Zivanovic, John Tracey, Adam S Foster, Angelika Kühnle

**Affiliations:** 1 Physical Chemistry I, Faculty of Chemistry, Bielefeld University, Universitätsstraße 25, 33615 Bielefeld, Germany; 2 Institute of Physical Chemistry, Johannes Gutenberg University Mainz, Duesbergweg 10 - 14, 55099 Mainz, Germany; 3 Graduate School Materials Science in Mainz, Staudingerweg 9, 55128 Mainz, Germany; 4 Department of Applied Physics, Aalto University, Helsinki FI-00076, Finland; 5 WPI Nano Life Science Institute (WPI-NanoLSI), Kanazawa University, Kakumamachi, Kanazawa 920-1192, Japan

**Keywords:** 3D AFM, calcite, ethanol, magnesite, MD simulation, solvation structure

## Abstract

Calcite and magnesite are important mineral constituents of the earth’s crust. In aqueous environments, these carbonates typically expose their most stable cleavage plane, the (10.4) surface. It is known that these surfaces interact with a large variety of organic molecules, which can result in surface restructuring. This process is decisive for the formation of biominerals. With the development of 3D atomic force microscopy (AFM) it is now possible to image solid–liquid interfaces with unprecedented molecular resolution. However, the majority of 3D AFM studies have been focused on the arrangement of water at carbonate surfaces. Here, we present an analysis of the assembly of ethanol – an organic molecule with a single hydroxy group – at the calcite and magnesite (10.4) surfaces by using high-resolution 3D AFM and molecular dynamics (MD) simulations. Within a single AFM data set we are able to resolve both the first laterally ordered solvation layer of ethanol on the calcite surface as well as the following solvation layers that show no lateral order. Our experimental results are in excellent agreement with MD simulations. The qualitative difference in the lateral order can be understood by the differing chemical environment: While the first layer adopts specific binding positions on the ionic carbonate surface, the second layer resides on top of the organic ethyl layer. A comparison of calcite and magnesite reveals a qualitatively similar ethanol arrangement on both carbonates, indicating the general nature of this finding.

## Introduction

Sedimentary rocks including the minerals calcite and magnesite are abundant constituents of the earth’s crust and their interaction with the environment is relevant for a wide range of geological processes, such as dissolution and weathering. Moreover, calcite plays a prominent role in many industrial processes, for example, incrustation inhibition and desalination. As any interaction between a mineral and its environment takes place at the interface, the interfacial structure is of fundamental importance for gaining molecular-level understanding of the mineral reactivity. In the presence of water, the hydration structure at the interface needs to be known. With the advent of high-resolution 3D atomic force microscopy (AFM) [1], the hydration structure of many interfaces has been studied, including the aqueous interface of mica [2] calcite [3–4] dolomite [5–6] and organic crystals [7]. However, while the majority of 3D AFM works have concentrated on water, comparatively fewer experimental studies exist addressing the interfacial arrangement of other solvent molecules [8–10]. This is unfortunate given the relevance of the interaction between organic molecules and carbonate surfaces, for example, in the field of biomineralization [11]. Moreover, by changing the solvent molecule, fundamental understanding can be gained when comparing the influence of systematically changed functional groups. Here, we focus on the arrangement of ethanol molecules as they have an OH group in common with water but differ in their properties due to their hydrocarbon chain.

The calcite–ethanol interface has been investigated theoretically by using both density functional theory (DFT) [12–13] and molecular dynamics (MD) simulations [14–17]. It has been found that ethanol molecules strongly bind towards calcite (10.4) terraces – even stronger than water [12,14,16–17]. Ethanol molecules bind towards calcite with their hydroxy group placed in between a surface calcium ion and a surface carbonate: The oxygen of the hydroxy group binds towards a calcium ion, while the hydrogen of the hydroxy group binds towards a carbonate group, which is similar to the bonding configuration of an isolated ethanol molecule on calcite obtained with DFT calculations [13]. Consequently, the hydrocarbon chains of the ethanol molecules point away from the surface. This results in one ethanol molecule per CaCO_3_ at the calcite (10.4) surface. The ordered first layer of ethanol molecules above the calcite surface is followed by a region of low ethanol density, which has been referred to as a gap [15,17]. Beyond the gap, ethanol again arranges in vertical layers with a vertical distance of approximately 0.5 nm. In contrast to the first layer, however, it has been calculated that both the lateral order and the orientational order of the ethanol molecules in the upper layers is significantly less pronounced. Thus, these theoretical studies predict that ethanol molecules at the calcite interface exhibit very different binding configurations depending in which layer they are.

Only isolated aspects of the above-mentioned theoretical studies have been confirmed experimentally. With lateral AFM images, the calcite (10.4) surface has been laterally resolved in ethanol at the atomic scale [18]. The observed periodicity matched the surface unit cell dimensions. However, from lateral AFM images it is not straightforward to determine at which vertical distance the tip was scanned above the surface. Therefore, it remains unclear whether the observed lateral structure corresponds to the first ordered layer of ethanol on calcite or the calcite surface itself.

The vertical structure of ethanol on calcite has been investigated in a combined X-ray reflectivity and MD study [15]. As a result, ethanol was found to form layers above the calcite surface. However, due to the lack of lateral resolution in the X-ray reflectivity measurements, no experimental information on the lateral order within the first layer has been obtained to date. Furthermore, no information on the interfacial orientation and binding configuration has been collected experimentally.

Here, we report on high-resolution 3D AFM data that reveals both the lateral and the vertical solvation structure at the calcite–ethanol interface in a single data set. By comparison with existing literature and MD simulations, we can assign the single laterally ordered layer to the first solvation layer. This first layer is fundamentally different from the following layers as the ethanol molecules adopt specific adsorption positions on the carbonate surface. In contrast, the second layer then resides on top of the less well-defined layer of ethyl groups. For the magnesite–ethanol interface, we find a remarkably similar solvation structure as for the calcite–ethanol interface in both the AFM data and the MD simulations. This latter result confirms the general nature of our findings.

## Results and Discussion

### AFM results

**Figure 1:**
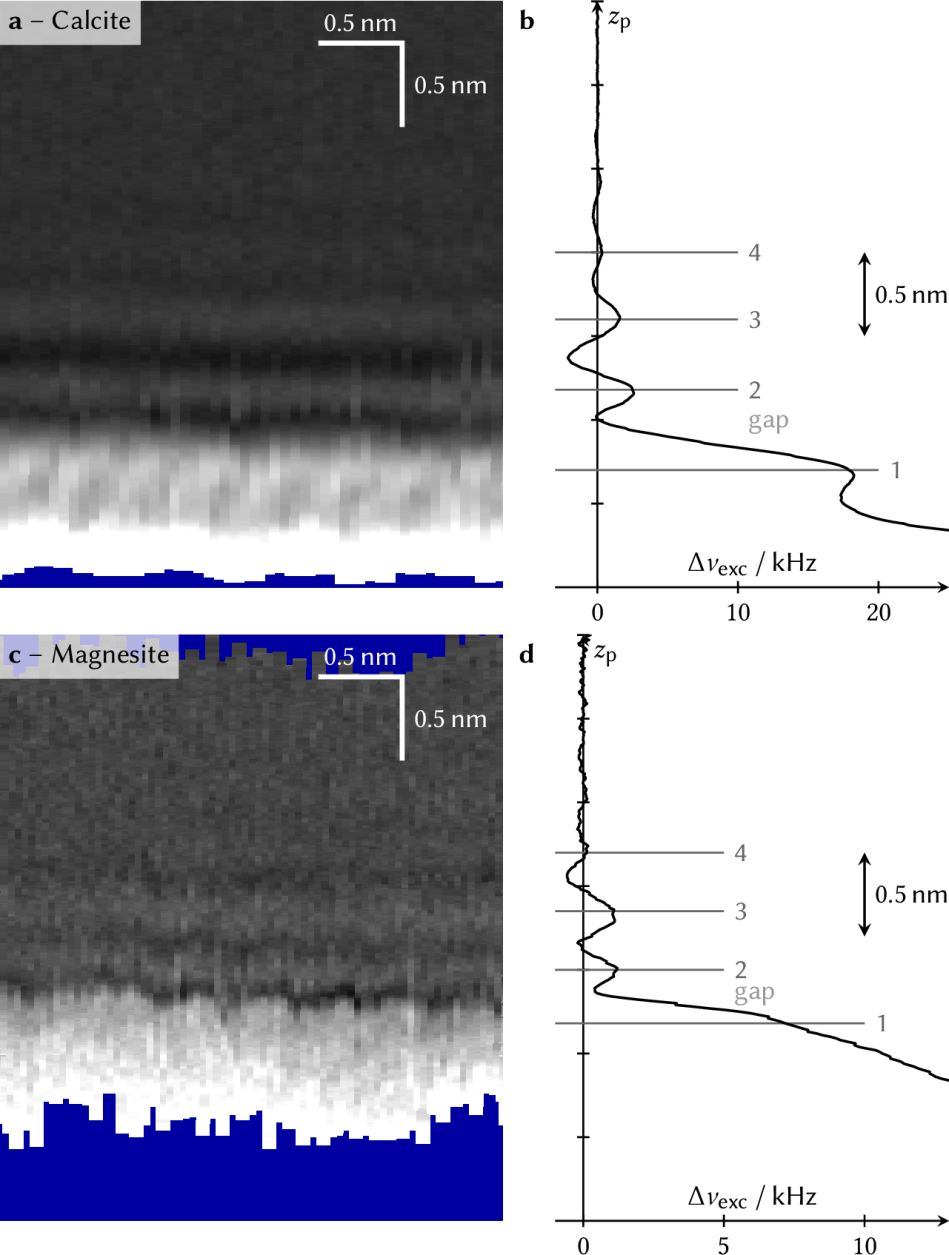
Vertical slice of the frequency shift at the calcite–ethanol interface (a) and at the magnesite–ethanol interface (c). The graphs on the right (b) and (d) show the average of all profiles shown in the slice. In all cases, the frequency is shown as a function of the *z*-piezo displacement *z*_p_. The black/white color scale ranges from approximately −5 kHz to 27 kHz for calcite and from approximately −5 kHz to 15 kHz for magnesite. The vertical scale bar in (a) and (c) equally applies to the profiles in (b) and (d).

A vertical slice of the frequency shift (Δν_exc_) obtained at the calcite (10.4)–ethanol interface is shown in Figure 1a. The average over all data shown in the slice is given as a vertical profile (i.e., as function of the *z*-piezo displacement *z*_p_) in Figure 1b. The corresponding data for the magnesite-ethanol interface is shown in a similar fashion in the second row of Figure 1 (panels c and d).

In both cases, the frequency shift exhibits local minima and maxima. Close to the surface (at the bottom), laterally alternating local maxima with a periodicity of approximately 0.3 nm can be observed. Note that due to lateral drift, it is not possible to quantify the lateral distance of the first-layer ethanol molecules with a precision that would allow discrimination between the different surface unit cell sizes of calcite and magnesite. Nevertheless, it appears very reasonable to assign this lateral structure to the ordered first layer of ethanol molecules. We note that the data of calcite and magnesite differ slightly when the tip is closer to the surface than the gap. While in the case of calcite a clear maximum (labelled 1 in Figure 1) is seen, the same feature appears to be a saddle point rather than a maximum in the case of magnesite. At this short tip–sample distance, the solvent–tip approximation [19–20] alone cannot explain the data, but instead the chemical nature and macroscopic shape of the tip plays a crucial role. Hence, the observed difference is likely due to the different tips used in the experiments.

The experimental data shows further layers above the first layer. In sharp contrast to the first layer, no lateral structure is visible in the upper layers. A precise determination of the absolute distances of the layers requires a precise calibration of the *z*-piezo movement, which is lacking for the presented data set. However, the comparison with previous images containing step edges allows determination of the vertical layer-to-layer distances that are in the order of 0.5 nm. This experimentally obtained layer-to-layer distance is in good agreement with the above-cited previous calculations of ethanol interfaces.

Moreover, observing no order in the upper layers fits well to the MD simulations from previous theoretical studies, which do not indicate significant lateral order in the layers above the first layer for the calcite–ethanol interface [14–17]. This finding is similar to what has been observed before for the solvation structure of 2-propanol on calcite (10.4) [3]. Also, for the latter work, lateral order was observed in the first layer only. Comparing the results obtained on calcite and magnesite reveals some minor differences that are within the variations typically seen for different tips. Thus, our experimental findings suggest these findings to be equally valid for the magnesite–ethanol interface.

### MD simulation results

**Figure 2:**
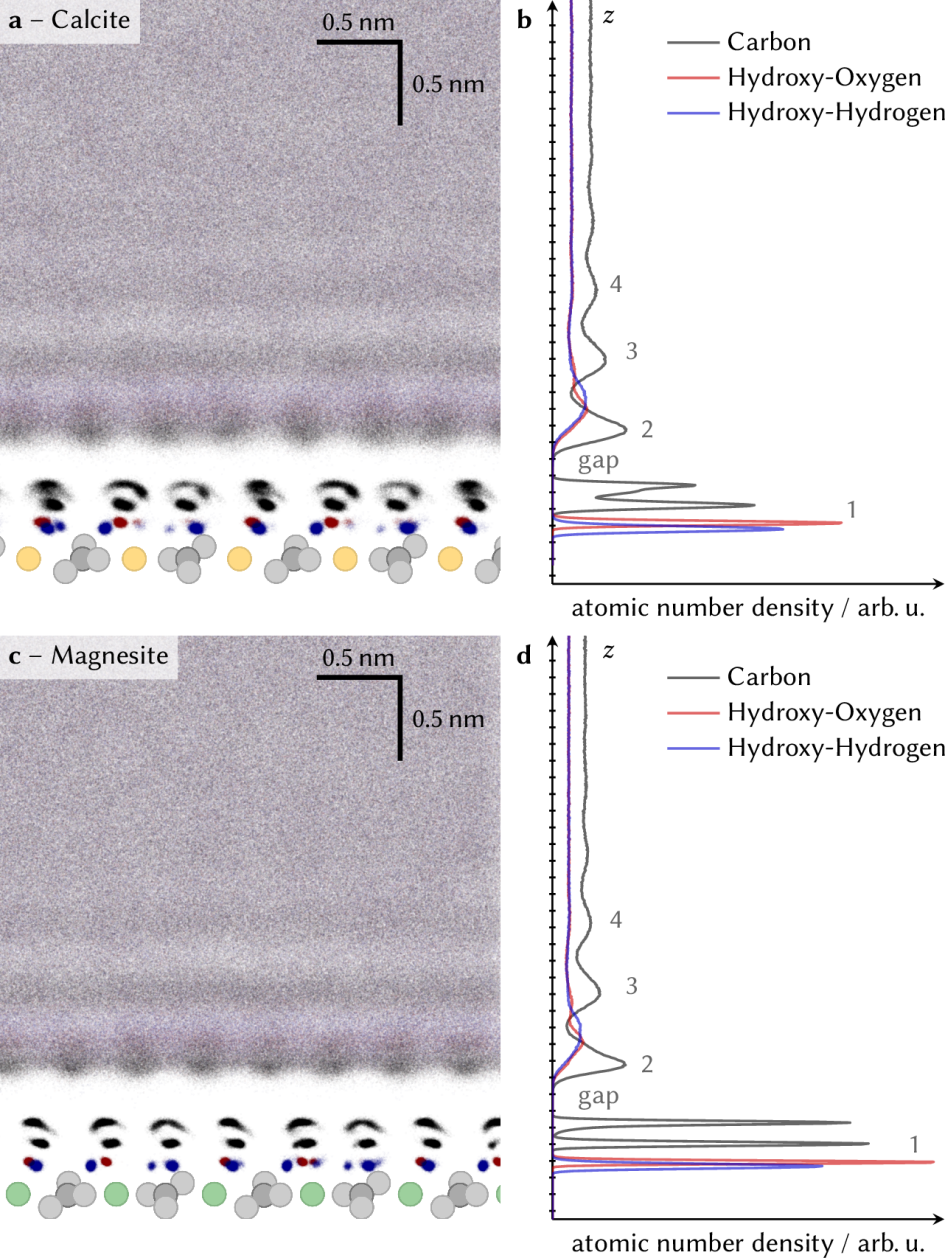
Vertical slices showing the atomic positions of ethanol–carbon (black), hydroxy–oxygen (red) and hydroxy–hydrogen (blue) at the (a) calcite–ethanol and (c) magnesite–ethanol interface. The graphs were created by drawing a semi-transparent dot for each atom at each time step of the simulation. The surfaces are represented by the carbonate groups (grey) and calcium (yellow) and magnesium (green) spheres, respectively. (b and d) Corresponding averaged density profiles are shown in the graphs to the right.

To allow for consistent comparisons between our experimental data and simulations, we performed MD simulations of both the calcite–ethanol and the magnesite–ethanol interface. In Figure 2 we present vertical slices and density profiles of both the calcite–ethanol (Figure 2a and 2b) and the magnesite–ethanol (Figure 2c and 2d) interface showing the atomic positions of the ethanol molecules throughout the production run of the simulation.

In the graphs in Figure 2b and 2d, the atomic number density profiles for ethanol–carbon, hydroxy–oxygen and hydroxy–hydrogen atoms averaged from the data presented in the slice are shown. The density maxima for the hydroxy atoms directly above the surface (at small *z*) indicate that the hydroxy group is oriented with the hydrogen towards the surface. The two distinguishable peaks for the carbon atoms following at larger distance from the surface show that the hydrocarbon chains of the molecules are all aligned perpendicular to the surface, pointing away from it. All atomic number density profiles show a pronounced minimum after this first layer of ethanol molecules on top of the calcite surface, the so-called “gap”. At larger distances from the surface, a second solvation layer can be identified by a peak in each of the atomic number density profiles. All three peaks in the second layer (carbon, oxygen, hydrogen) are significantly broader compared to the first layer. In sharp contrast to the first solvation layer, where the hydroxy group is oriented towards the surface, ethanol in the second layer shows only a very weak orientational preference (there is a slight preference for the OH to point away from the surface).

Even broader peaks can be recognized in the profiles at larger distances from the surface, which is indicative of a faint third, fourth, etc. layer. From the MD simulation, we obtain a layer-to-layer distance of approximately 0.5 nm, which fits well with the experimental AFM data.

**Figure 3:**
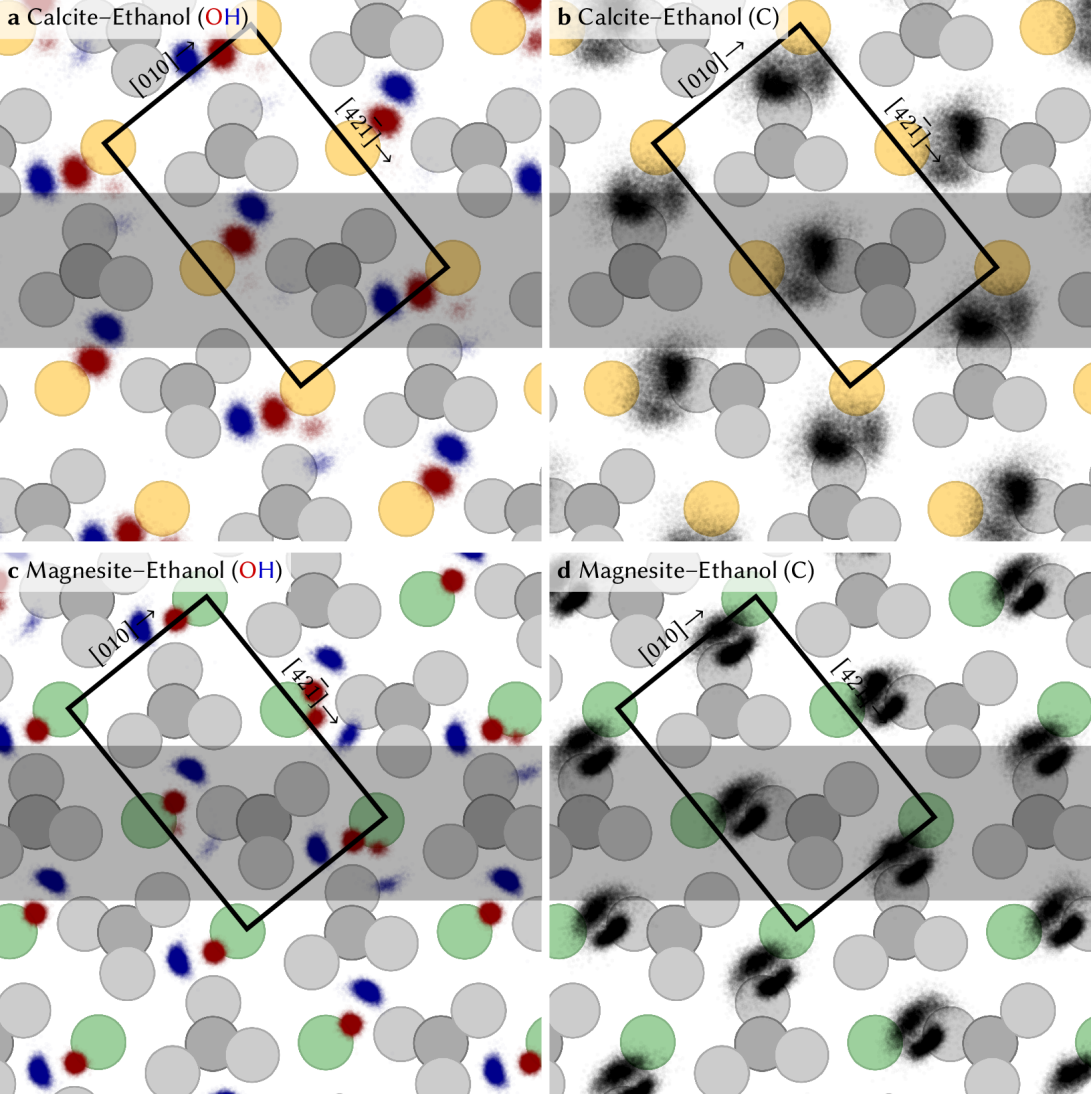
Top view showing the position of all hydroxy–oxygen (red) and hydroxy–hydrogen (blue) atoms on (a) calcite and (c) magnesite as well as the position of all carbon atoms (black) of ethanol molecules within the first layer on (b) calcite and (d) magnesite in each simulation time step. The grey overlay indicates the area used for the vertical slice in Figure 2. The positions of the surface atoms are indicated by grey, yellow and green spheres, representing atoms from the carbonate groups, as well as calcium and magnesium atoms, respectively.

To further investigate the lateral order in the first layer, we show the lateral hydroxy–oxygen density within the first solvation layer, superimposed on a lateral view of the calcite and magnesite surfaces in Figure 3. The hydrogen and oxygen positions at the calcite–ethanol interface are given in Figure 3a, while the positions of all carbon atoms in the first layer are shown in Figure 3b. The respective information for the magnesite–ethanol interface is presented in Figure 3c and Figure 3d. The OH-oxygen is preferably located in between a calcium and a carbonate ion. This is in accordance with the previous MD simulations [14–17], which have shown that ethanol arranges in a ordered first layer above calcite, where one ethanol molecule binds towards one CaCO_3_ unit. The oxygen is closer to the cation (Ca/Mg) and the hydrogen creates a hydrogen bond with a protruding oxygen of a carbonate group. During the time of the simulation (10 ns), all ethanol molecules in the first layer exhibit a highly confined lateral position, which fits to the observed lateral order. In rare cases, we observed that some of the hydrogens from the OH group switch the hydrogen bond back and forth to a neighboring carbonate group within a very similar distance during the simulation run, which we further discuss in Supporting Information File 1.

To compare the MD simulations with the AFM data, we use the well-established “solvent–tip approximation” [19–20]. We note that it is relevant to discuss whether this approximation holds true for ethanol as well. However, in this work, we assume that a single ethanol molecule probes the solvation structure. In this model, the frequency shift modulation is approximately proportional to the solvent density. This model has worked well in previous works with the solvent water [10], even in the case of defects [21].

There is excellent qualitative agreement between the experimentally obtained frequency shift data (Figure 1) and the atomic density data from the MD simulations (Figure 2). The AFM data confirms the laterally alternating minima and maxima within the first layer and the oscillatory vertical density profile predicted by the MD simulations.

## Conclusion

We combined high-resolution 3D AFM with MD simulations to characterize the solvation structure of ethanol above calcite and magnesite (10.4) surfaces. For the calcite surface, our high-resolution AFM data revealed a layered structure. In the first layer, a lateral structure is visible in the data. The layers above the first layer, however, do not show any lateral structure.

Therefore, the experimental data indicate that ethanol molecules form a single laterally ordered layer directly above the calcite surface, which fits perfectly to the MD simulation data. This finding can be understood by the different chemical environment. While the first layer adopts well-defined adsorption positions on the carbonate surface, the following layers only reside on ethanol layers with clear consequences for the lateral order. For a structurally very similar system, namely the magnesite–ethanol system, we find that both the AFM data as well as the MD data are similar to the calcite data. This latter result indicates that the finding made here is of general nature.

## Experimental and Theoretical Methods

### Atomic force microscopy

For the AFM experiments we used a modified commercial atomic force microscope [22] with custom photothermal cantilever excitation [23] and a custom three-dimensional scanning and data acquisition mode [24] in the frequency-modulation mode [25]. The quantities given in this work are labelled as introduced in [26]. After cleaving calcite (Korth Kristalle, Germany) and magnesite (SurfaceNet, Germany) in air, ethanol (Sigma Aldrich, article number 32205, purity ≥ 99.8%) was injected in the liquid cell. Since the ethanol was exposed to air during the measurement, it constantly evaporates, making it necessary to repeatedly inject ethanol during a measurement session. Cantilevers of type TAP300 GB-G were used. The acquisition time for each vertical slice (trace and retrace) of a 3D map was 10 s and the frequency of the (approach and retract) *z*-modulation was 10 Hz, corresponding to 50 approach and 50 retract curves per vertical slice. For typical operation parameters, we refer to our earlier work [5,24,26].

While we show frequency shift data in the main text, we include a detailed discussion on the static deflection in Supporting Information File 1, where we also discuss the robustness and reproducibility of the AFM results.

### Molecular dynamics simulations

We employed molecular dynamics simulations to model both calcite and magnesite with their (10.4) surface exposed as a nine layer crystal with three surface unit cells in the 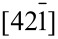 direction and six surface unit cells in the [010] direction. Each crystal was first centered in the simulation box. The vertical box size (in *z*-direction) was then increased up to 15 nm in order to place ethanol molecules on both exposed (10.4) surfaces, thus ensuring bulk properties of the solvent far away from the surface. The obtained systems, composed of 1620 crystal atoms and 800 ethanol molecules, are charge neutral and periodic in all three directions. The surface unit cell of the simulated crystal measured 0.81 × 0.49 nm^2^ for calcite and 0.73 × 0.46 nm^2^ for magnesite.

We first performed an energy minimization of the system with conjugated gradients [27–28] to ensure that the atoms from either ethanol or mineral were in an initially stable configuration. After that, an equilibration of 0.5 ns in an NVT ensemble was performed, followed by 5 ns equilibration in an NPT ensemble and another 2 ns in a NVT ensemble. The production run was performed after that in an NVT simulation of 10 ns.

Temperature control in NVT and NPT ensembles was set to 300 K, using the Nosé–Hoover scheme [29–31] with five thermostat chains. In the NPT ensemble, pressure control was set to 1 atm using Nosé–Hoover barostats [29]. Electrostatics were calculated with the particle-particle-particle-mesh method [32].

In order to prevent drift of the entire system, the carbon atoms in the crystal middle layer were kept fixed during the dynamics. A time step of 1 fs was used for the integration of the equation of motion, ensuring proper energy conservation.

The output data was collected every 1 ps during the production run, providing enough statistics for all required analysis. MD simulations were performed in Lammps code [33]. The analysis was performed using the Python library MDAnalysis [34–35].

Calcite and magnesite were described by the force-field developed in [36–37]. It has been shown in previous calcite studies that this force field successfully describes all the calcite properties. Ethanol was described by the CHARMM General Force Field (CGenFF) [38–39]. The cross-terms between the minerals and ethanol were obtained through Lorentz–Berthelot combination rules [40].

## Supporting Information

File 1 Additional information.
