# Supplementary material for: Three-dimensional solvation structure of ethanol on carbonate minerals
Source: Beilstein J Nanotechnol. 2020 Jun 10;11:891–8. doi: 10.3762/bjnano.11.74 (PMC7296196; doi:10.3762/bjnano.11.74)
Supplement: File 1 — Additional information. [file Beilstein_J_Nanotechnol-11-891-s001.pdf]

BEILSTEIN JOURNAL OF NANOTECHNOLOGY

Supporting Information

for

Three-dimensional solvation structure of ethanol on carbonate minerals

Hagen Söngen, Ygor Morais Jaques, Peter Spijker, Christoph Marutschke, Stefanie Klassen, Ilka Hermes, Ralf Bechstein, Lidija Zivanovic, John Tracey, Adam S. Foster and Angelika Kühnle

Beilstein J. Nanotechnol. **2020,** *11,* 891–898. doi:10.3762/bjnano.11.74

Additional information

License and Terms: This is a supporting information file under the terms of the Creative Commons Attribution License (http://creativecommons.org/licenses/by/4.0). Please note that the reuse, redistribution and reproduction in particular requires that the authors and source are credited.
The license is subject to the *Beilstein Journal of Nanotechnology* terms and conditions: (https://www.beilstein-journals.org/bjnano)

Discussion of the static deflection

While we consistently observed the vertically-layered solvation structure, the observation of the laterally-ordered first solvation has been significantly less frequent. A possible reason for this difficulty (in comparison to water, where laterally ordered structures are routinely observed) might be the stronger bond of the first ethanol layer towards the substrate surfaces.

A valid question is whether we can ensure that we actually reached the sample surface with the cantilever tip. While a definitive answer is outside the scope of atomic force microscopy, a reasonable indication is to test whether the cantilever has been approached towards the sample as far as possible. In Figure S1 we present additional data that shows the vertical photo diode voltage (as an indication of the static deflection q_s divided by the static deflection sensitivity s of the cantilever) and the corresponding excitation frequency during each individual cantilever approach and retract while obtaining the vertical slice shown in Figure 1 of the main text. Both quantities are shown as function of the z-piezo displacement z_p. To facilitate an overview of the individual curves, we shifted each individual approach and retract profile such that the value at the outer turning point (at the largest z-piezo displacement) is zero. This was necessary, as the vertical photo diode voltage drifted during the measurement.

In the case of calcite, the static deflection increases at small z-piezo displacements, indicating that the actual tip–sample distance does not decrease (instead, the cantilever just bends). For the shown magnesite data we see this decrease of the static deflection along with a major hysteresis. In other data sets, we also observe such a hysteresis at the calcite-ethanol interface. The origin of this hysteresis is unknown.

Radial distribution function

We calculated the radial distribution function between two atoms of species a and b using:

$$g_{ab}(r) = \frac{V}{N_a N_b} \sum_{i=1}^{N_a} \sum_{j=1}^{N_b} \langle \delta(|\mathbf{r_i} - \mathbf{r_j}| - r) \rangle \tag{S1}$$

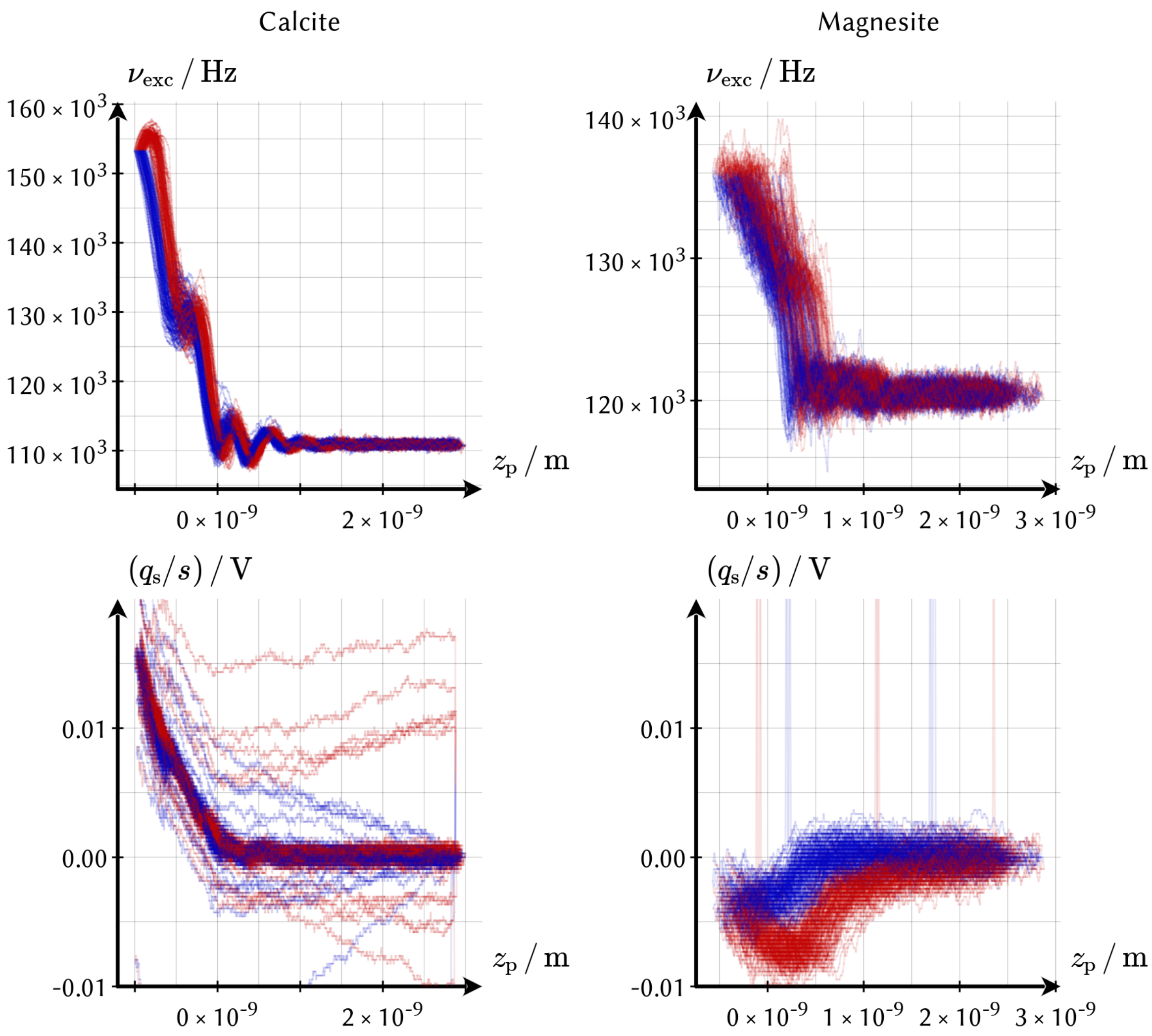

Figure S1: Comparison of the excitation frequency (top row) with the static deflection raw data (bottom row) for calcite (left) and magnesite (right). Data for the tip approach is shown in blue, data obtained during the tip retract is colored red.

The above expression sums up the number of neighboring atoms of type b around each of the atoms of type a in a sphere of radius r centered in each atom. The total number of atoms are N_a and N_b, respectively.

The coordination number is calculated through the expression:

$$N_{ab}(r) = \rho \int_0^r dr' 4\pi r'^2 g_{ab}(r') \qquad \text{(S2)}$$

where ρ is the local density of the sphere of radius r.

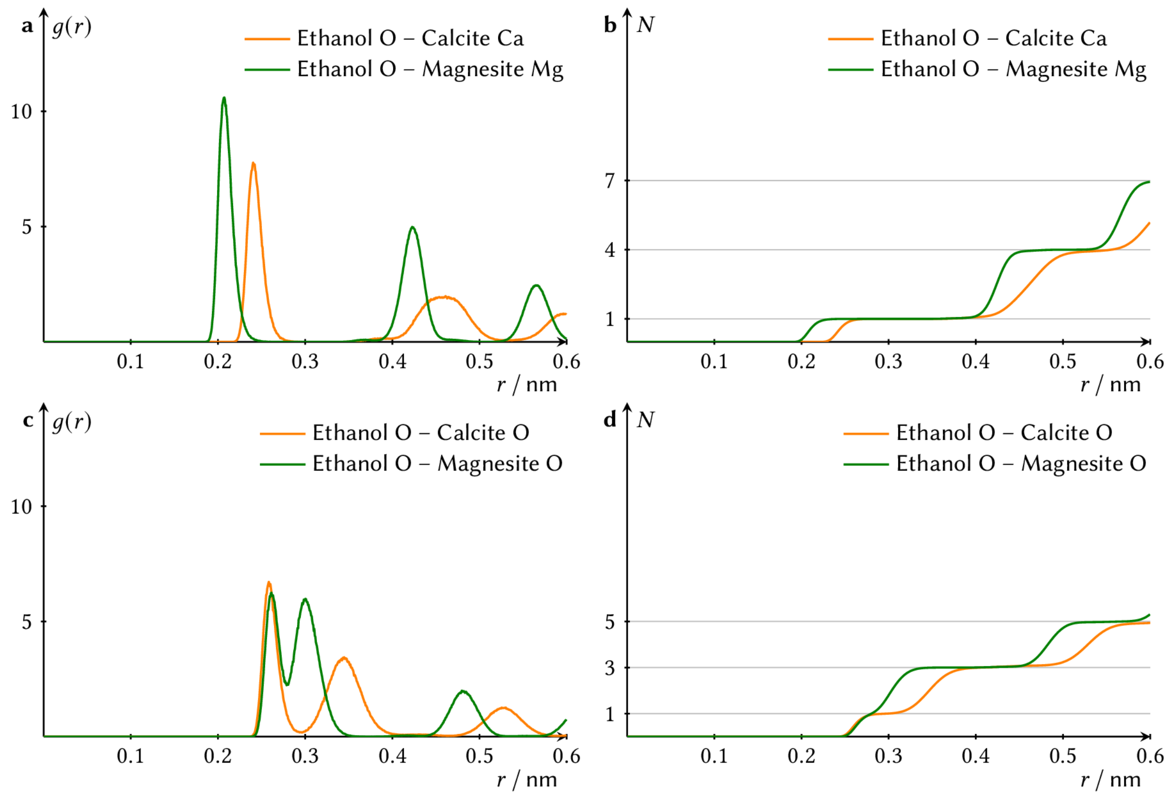

Figure S2: Radial distribution function (a) and coordination number (b) between the oxygen atoms of the ethanol hydroxy group and the surface cations. Radial distribution function (c) and coordination number (d) between the oxygen atoms of the ethanol and the surface oxygen atoms.

Atomic coordination between ethanol and carbonate surfaces

Most of the time, the hydrogen bonds formed between ethanol and carbonate groups occur in a ordered fashion, with one hydrogen interacting with one specific protruding oxygen from a carbonate group, as shown in Figure 2 of the main text. In both calcite and magnesite lattices, the distance between Ca/Mg and the first protruding oxygen from CO_3 is within 0.20–0.25 nm, being to short for a hydrogen bond. However, the second and third-closest protruding oxygens are within a distance of 0.30–0.35 nm, allowing hydrogen bond formation. The small difference in the distance between the cation and these two neighboring oxygens results in a flipping of the ethanol OH groups, that at each step of the dynamics can be either closer to one or the other.

Even though the flipping of OH groups occurs for both carbonate minerals, we observed some differences between them. The density of ethanol oxygen is more concentrated on magnesite than

on calcite. This is due to a stronger interaction of the oxygen with the Mg in relation to Ca ions, that can be seen in the radial distribution function $g(r)$ shown in Figure S2a. This restrains the hydroxy-oxygen movement on magnesite relative to the calcite surface. However, the number of atoms in both structures are the same in the first solvation layer, as can be seen in Figure S2b, showing one ethanol oxygen per cation.

Furthermore, the OH-flipping is more pronounced on calcite compared to m agnesite. This is due to the larger unit cell of calcite in relation to magnesite. In Figure S2c we can see that the first protruding oxygen of CO_3 that can bind towards the ethanol oxygen is located within approximately the same distance in both calcite and magnesite. However, for the second (rarer) possible coordination, magnesite presents a closer oxygen than calcite, making the flipped c onfiguration le ss st able. In calcite, however, the larger distance between the two oxygen makes a particular configuration (flipped or non-flipped) to be maintained for longer. This difference appears more clearly when we count the ratio of flipped and non-flipped configurations. The flipped configuration occurs in about 6.1% of the time for calcite and in 3.9% of the time for magnesite. Figure S2d shows that the same coordination numbers are obsterved in both structures.
